# The Plastome Sequences of *Triticum sphaerococcum* (ABD) and *Triticum turgidum* subsp. *durum* (AB) Exhibit Evolutionary Changes, Structural Characterization, Comparative Analysis, Phylogenomics and Time Divergence

**DOI:** 10.3390/ijms23052783

**Published:** 2022-03-03

**Authors:** Lubna, Sajjad Asaf, Rahmatullah Jan, Abdul Latif Khan, Waqar Ahmad, Saleem Asif, Ahmed Al-Harrasi, Kyung-Min Kim, In-Jung Lee

**Affiliations:** 1 Department of Botany, Garden Campus, Abdul Wali Khan University, Mardan 23200, Pakistan; lubnabilal68@gmail.com; 2 Natural and Medical Sciences Research Center, University of Nizwa, Nizwa 616, Oman; sajadasif2000@gmail.com (S.A.); waqarahmed111@gmail.com (W.A.); aharrasi@unizwa.edu.om (A.A.-H.); 3 Division of Plant Biosciences, School of Applied Biosciences, College of Agriculture & Life Science, Kyungpook National University, Daegu 41566, Korea; rehmatbot@yahoo.com (R.J.); saleemasif10@gmail.com (S.A.); kkim@knu.ac.kr (K.-M.K.); 4 Department of Engineering Technology, College of Technology, University of Houston, Sugar Land, TX 77479, USA

**Keywords:** wheat, polyploidy, chloroplast genome, inverted repeats, *Triticum sphaerococcum*, *Triticum turgidum* subsp. *durum*

## Abstract

The mechanism and course of *Triticum* plastome evolution is currently unknown; thus, it remains unclear how *Triticum* plastomes evolved during recent polyploidization. Here, we report the complete plastomes of two polyploid wheat species, *Triticum sphaerococcum* (AABBDD) and *Triticum turgidum* subsp. *durum* (AABB), and compare them with 19 available and complete *Triticum* plastomes to create the first map of genomic structural variation. Both *T. sphaerococcum* and *T. turgidum* subsp. *durum* plastomes were found to have a quadripartite structure, with plastome lengths of 134,531 bp and 134,015 bp, respectively. Furthermore, diploid (AA), tetraploid (AB, AG) and hexaploid (ABD, AGA^m^) *Triticum* species plastomes displayed a conserved gene content and commonly harbored an identical set of annotated unique genes. Overall, there was a positive correlation between the number of repeats and plastome size. In all plastomes, the number of tandem repeats was higher than the number of palindromic and forward repeats. We constructed a *Triticum* phylogeny based on the complete plastomes and 42 shared genes from 71 plastomes. We estimated the divergence of *Hordeum vulgare* from wheat around 11.04–11.9 million years ago (mya) using a well-resolved plastome tree. Similarly, Sitopsis species diverged 2.8–2.9 mya before *Triticum urartu* (AA) and *Triticum monococcum* (AA). *Aegilops speltoides* was shown to be the maternal donor of polyploid wheat genomes and diverged ~0.2–0.9 mya. The phylogeny and divergence time estimates presented here can act as a reference framework for future studies of *Triticum* evolution.

## 1. Introduction

Triticeae Dumort. is an economically valuable grass tribe with around 360 species and subspecies in 20–30 genera. The genus *Triticum* is an important agricultural allopolyploid complex containing two diploids, two tetraploids, and two hexaploids. Over 10,000 years, one diploid and both tetraploid species have been domesticated, whereas two hexaploid species emerged under cultivation in Eurasia [1]. Hybridization of a cultivated type of tetraploid *Triticum turgidum* (AABB genomes) with diploid goat grass *Aegilops tauschii* (DD genome) resulted in hexaploid common wheat, i.e., bread wheat (*Triticum aestivum*, AABBDD genomes). Similarly, *Triticum zhukovskyi* (AAGGA^m^A^m^ genomes), the second hexaploid wheat, was produced by crossing tetraploid Timopheevi wheat (*Triticum timopheevii*, AAGG genomes) with diploid einkorn (*Triticum monococcum* ssp. *monococcum*, A^m^A^m^ genome). The Emmer lineage of wheat includes *T. turgidum* and *T. aestivum*, whereas the Timopheevi lineage includes *T. timopheevii* and *T. zhukovskyi* [2]. *Triticum urartu* (genome AA) contributed the A genome to the Emmer and Timopheevi lineages as the male parent [3,4], whereas *Aegilops speltoides* (SS genome) contributed the cytoplasm [5] and G genome [6,7] to *T. timopheevii* as the female parent. Since Jenkins initially identified *A. speltoides* as a probable donor for the B genome and the cytoplasm of *T. turgidum* 90 years ago [8], the origin of the B genome and the cytoplasm of *T. turgidum* has been disputed. Previous research has proposed that the B genome came from one of the other diploid Sitopsis species in the genus *Aegilops*, i.e., *Aegilops searsii* (S^s^ S^s^ genome), *Aegilops sharonensis* (S^sh^ S^sh^ genome), *Aegilops longissima* (S^l^ S^l^ genome), or *Aegilops bicornis* (S^b^ S^b^ genome) [9].

Low barriers to hybridization are known to exist in Triticeae, which has resulted in mixed or even recombinant phylogenetic evidence from nuclear data [10,11]. In Triticum and the closely related genus Aegilops, chloroplast DNA is inherited maternally [12]; consequently, chloroplast evolution has been efficiently and critically investigated in Triticum and *Aegilops* with the aim of clarifying the genetic variety of the cytoplasm as well as the maternal lineage [13]. Organelles are typically uniparentally inherited and nonrecombining in angiosperms; hence, phylogenetic studies of chloroplast genomes offer unequivocal information on maternal lineages [14]. However, chloroplast capture [15] can result in conflicting phylogenetic assumptions. Currently, there is a scarcity of plastome sequencing information for Triticeae. Only a few studies [16,17,18,19,20] have employed a tribe-wide taxon sample and they have focused on a single plastome or a few plastomes markers. Although the number of full plastome sequences is growing [21,22,23,24,25,26,27,28], whole plastomes are mostly available for domesticated species and their near relatives. Because just one to a few accessions per taxon have been included in earlier research and given that support values for taxonomic units have generally been low, prior analyses have provided only limited insights into the maternal phylogeny of Triticeae [17,19,29].

Typically, plastomes are 115–165 kb in size [30]. Plastomes of the Poaceae family have a similar structure, with large single copy (LSC) and short single copy (SSC) regions of about 80 and 13 kb in length, respectively, placed between two inverted repeat sequences with lengths of about 20 kb [31,32,33]. Different genes of the nuclear genome and plastome appear to have different genealogical histories; however, it is not known whether the historical connections among the genera and species of this tribe can be resolved in a tree-like manner. If so, the true phylogenetic relationships remain unclear because published trees are in conflict for almost all taxon positions [10].

In the current study, we sequenced the complete plastomes of two *Triticum* species, *T. sphaerococcum* (AABBDD) and *T. turgidum* subsp. *durum* (AABB), and compared them with 19 complete *Triticum* plastomes available in NCBI database (1 June 2021). We intended to answer the following key questions about *Triticum* plastomes. (1) What are the common features of *Triticum* plastomes (such as gene order, gene content, gene gain, gene loss, and genome size variation)? (2) Elucidate the factors that influence the size of *Triticum* plastomes? (3) Based on the whole plastome and genes shared among the plastomes, what was the estimated divergence time of all *Triticum* available species? (4) To determine variation in the types of repeats found in these plastomes? Overall, our results will help elucidate the evolution of *Triticum*, as well as build a better taxonomy for this economically important genus.

## 2. Material and Methods

### 2.1. Sequencing, Assembly and Annotation

Fresh young leaves of *T. sphaerococcum* (AABBDD) and *T. turgidum* subsp. *durum* (AABB) were collected from plants growing in Swat region of Khyber Pakhtunkhwa province, Pakistan. The plants were identified by lead taxonomist at National Agricultural Research Centre (NARC), Pakistan. Both species, *T. sphaerococcum* and *T. turgidum* subsp. *durum* are available with accesion number 3 and 25, respectively at Gene Bank of Bioresources Conservation Institute, Pakistan. The leaf samples were collected in plastic zip bags and immediately kept in liquid nitrogen and stored at −80 °C for further analysis. A standard protocol for DNA extraction was followed, as described previously [34]. Pure DNA was sequenced using an Illumina HiSeq2000. In total, 115,455,321 and 86,123,254 raw reads were generated for *T. sphaerococcum* and *T. turgidum* subsp. *durum*, respectively. Next, the quality of paired-end Illumina reads was assessed in FastQC and the pipeline GetOrganelle version 1.6.2. [35] was used to select trimmed reads with default settings that corresponded to the plastid using the plastome of *T. aestivum* as a reference. Finally, the plastid-filtered reads from GetOrganelle version 1.6.2 were imported into Geneious Prime using the default settings. Furthermore, the complete plastomes of 19 *Triticum* species available in GenBank (as of 18 May 2021) were downloaded from the NCBI database (Table S1). The species with incorrect annotations were reannotated using CpGAVAS [36] and DOGMA [37] (http://dogma.ccbb.utexas.edu/ accessed on 25 February 2022, China). Moreover, tRNAscan-SE version 1.21 [38] was used to detect tRNA genes. Finally, the annotations were verified by Geneious Prime [39]. Graphical presentations were completed in R 4.0 and the ggplot2 package [40]. The number of genes shared among *Triticum* plastomes was identified via a Venn diagram webtool (bioinformatics.psb.ugent.be/webtools/Venn/ accessed on 25 February 2022). Pearson’s correlation coefficients were used to evaluate the associations among different plastomes characteristics, and the graphs were illustrated using R software (https://www.r-project.org/ accessed on 25 February 2022) cor function.

### 2.2. Characterization of Repetitive Sequences and SSRs

REPuter was used to determine the repetitive sequences (direct, reverse, and palindromic repeats) within plastomes [41]. For repeat identification, the following settings were used in REPuter: (1) a minimum repeat size of 30 bp, (2) ≥90% sequence identity, and (3) a Hamming distance of 1. Tandem Repeats Finder version 4.07b was used to find tandem repeats with the default settings applied [42]. To find SSRs, MISA [43] was employed with the search parameters set to ≥3 repeat units for pentanucleotide and hexanucleotide repeats, ≥4 repeat units for trinucleotide and tetranucleotide repeats, ≥8 repeat units for dinucleotide repeats, and ≥10 repeat units for mononucleotide repeats.

### 2.3. Sequence Divergence, Phylogenetic Analyses, and Divergence Time

For genome divergence among *Triticum* plastomes, mVISTA [44] in Shuffle-LAGAN mode was used with *T. turgidum* subsp. *durum* selected as a reference genome. The complete plastome and 42 shared protein-coding genes sequence divergence among *Triticum* species was calculated. Comparative analysis was performed after multiple sequence alignment and gene order was compared to identify ambiguous and missing gene annotations. To align the complete plastomes, MAFFT version 7.222 [45] was used with the default parameters and Kimura’s two-parameter (K2P) model [46] was used to determine pairwise sequence divergence. To resolve the phylogenetic position of *Triticum*, the complete plastomes and 42 shared genes from 71 (including 23 complete and 39 draft plastomes) of *Triticum* were used for analysis. Initially, a separate ML analysis of these data were conducted using RAxML [47] implemented in CIPRES with the default general time reversible (GTR + G) model and the fast bootstrap option, as previously reported by [48]. The resulting phylogenetic reconstruction was displayed using FigTree version 1.4.1 [49] and Interactive Tree Of Life (iTOL) version 6 [50].

To determine the divergence time of the *Triticum* genus relative to those of Triticeae species, we used a concatenated data matrix. Briefly, the GTR + G substitution model was used with four rate categories and a Yule tree speciation model was applied with a lognormal relaxed clock model in BEAST [51] using a prior rate of substitution. We used an average substitution rate of 3.0 × 10^−9^ substitutions per site per year (s/s/y) and a fossil-based method to calibrate the molecular divergence as previously reported [52]. To root the calibration time, we included four outgroup *Hordeum* species: *H. vulgare* subsp. *vulgare*, *H. vulgare* subsp. *spontaneum*, *H. jubatum*, and *H. bogdani*. We also incorporated four fossil constraints (Supplementary Table S2) that are widely recognized and have been used in previous molecular dating of Triticeae [53]. The mean root height constraint of 11.8 ± 1.8 mya was based on the work of [53,54]. This calibration is consistent with previous work completed by [22]. We selected these outgroups as all these species are closely related to our study model species and have fossil records older than that of the *Triticum* genus [53].

Calibration limited to the root height was used to compare with internal calibrations and assess their influence on divergence time estimates. The dating analyses involved three independent Markov Chain Monte Carlo runs of 25 million generations. LogCombiner was used to combine the tree files from each of the three runs. Convergence and effective sample sizes were assessed in Tracer 1.5 [55]. From each analysis, we removed 25% of trees as burn-in. Finally, the tree was calculated using TreeAnnotator and a tree with the 95% highest posterior density was visualized in FigTree 1.4.

## 3. Results

### 3.1. Triticum Plastome Characteristics (Genome Size and GC Content Variation)

**Figure 1:**
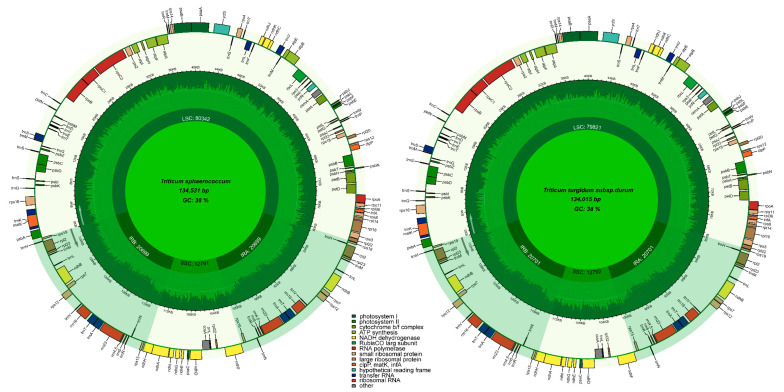
Genome map of the *T. sphaerococcum* and *T. turgidum* subsp. *durum* plastomes. The extent of the IR regions is represented by dark colors, which divide the cp genome into large (LSC) and small (SSC) single copy regions. Genes drawn inside the circle are transcribed clockwise, whereas those outside of the circle are transcribed counter-clockwise. Genes belonging to different functional groups are color coded. The light green in the inner circle corresponds to the GC content, whereas the dark green corresponds to the AT content.

The complete chloroplast genomes (cp) of the two sequenced Triticum species, *T. sphaerococcum* (MZ230675), *T. turgidum* subsp. *durum* (MZ230674), are circular molecules like typical angiosperm cp genomes having quadripartite structures. The sizes of the *T. sphaerococcum* and *T. turgidum* subsp. *durum* plastomes are 134,015 bp and 134,531 bp, respectively (Figure 1). Both *T. sphaerococcum* and *T. turgidum* subsp. *durum* plastomes were analyzed and compared with 19 associated *Triticum* cp genomes, with sizes ranging from 133,873 bp (*T.* *aestivum;* KJ592713) to 136,886 bp (*T. monococcum* subsp. *monococcum* (LC005977)) (Table 1). Even in the sequenced plastomes of similar species reported by various authors, variation in plastome length was detected in *Triticum* species. Both *T. sphaerococcum* and *T. turgidum* subsp. *durum* species in this study, had a pair of IR regions 20,699 and 20,701 bp, which divided the LSC region from the SSC, respectively, whereas the LSC and SSC lengths in *T. sphaerococcum* (80,342 and 12,791) and *T. turgidum* subsp. *durum* (79,817 and 12,788 bp). Both of these species had the same GC content in their genomes, 38.3% in the whole plastome and 44% in the IR regions (Figure 1, Table 1).

### 3.2. Gene Content and Gene Loss in Triticum Plastomes

**Figure 2:**
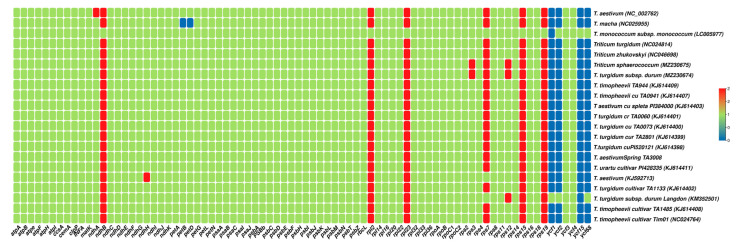
Summary of genes lost across *Triticum* plastomes. Blue color shows the missing genes, whereas the red color shows the genes duplicated in plastomes.

The gene content of the 21 *Triticum* plastomes varied considerably. There were 72–89 protein-coding genes, 8 rRNA genes, and 32–42 tRNA genes in these plastomes (Figure 1, Table 1). The number of genes annotated in a plastome ranged from 112 (*T. macha*, *T. monococcum* subsp. *monococcum*) to 136 (*T. turgidum* subsp. *durum* cultivar Langdon). Both the plastomes sequenced in this study had 131 genes including 8 rRNA genes, 39 tRNA genes, and 84 protein-coding genes (5 genes for photosystem I, 15 genes associated with photosystem II, 11 genes for large ribosomal proteins, and 17 genes related to small ribosomal proteins) (Table 2). In both plastomes, the lengths of the protein-coding region, tRNA, and rRNA were 59,538, 3004, and 9192 bp, respectively (Table 1). The gene contents were generally conserved throughout all *Triticum* species (Figure 2). The *ycf1*, *ycf2*, *ycf15*, and *ycf68* genes were lacking in almost all plastomes (Figure 2), with the exception of the *T. turgidum* subsp. *durum* langdon plastome in which the *ycf1* gene was detected. Notably, as in previous plastomes, the plastid gene *accD* was lost in all *Triticum* plastomes. The *rps12* gene (small ribosomal protein 12) is trans spliced and has one intron; the 5′ end exon is in the LSC region, whereas the 3′ end exon is in the IRb regions and duplicated in the IRa region.

Each of the sequenced *Triticum* species contained 12 intron-containing protein genes and 8 tRNA genes. Similar to in other angiosperm plastomes, *ycf3* contained two introns, whereas the remaining genes had only a single intron while the *rps12* gene is trans-spliced. The smallest introns were found in the *trnS-CGA* gene in both *T. sphaerococcum* and *T. turgidum* subsp. *durum* (658 bp), whereas the largest intron-containing gene was found in *trnK*-UUU in *T. sphaerococcum* (2486 bp) and in *T. turgidum* subsp. *durum* (2490 bp) (Table 3). The protein-coding region formed 44.2% of the whole plastome of *T. sphaerococcum* and 44.4% of that of *T. turgidum* subsp. *durum*. Similarly, tRNA and rRNA respectively comprised 2.23% and 6.83% of former species and 2.24% and 6.85% of the latter species.

### 3.3. Functional Repeats within Triticum Plastomes

**Figure 3:**
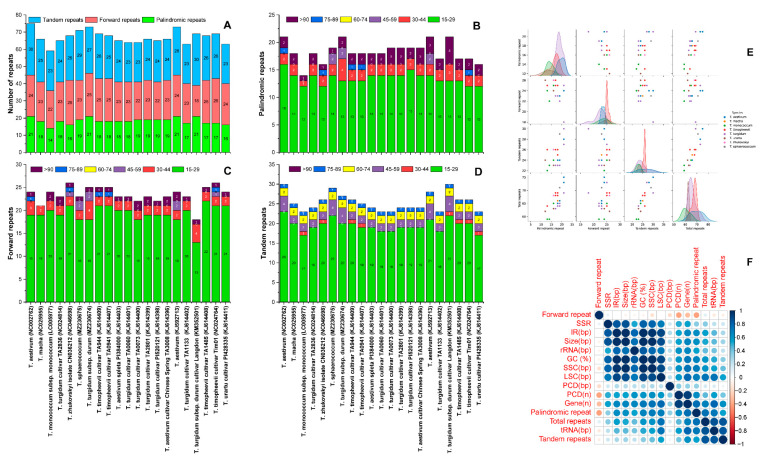
Analysis of repeated sequences in the 21 *Triticum* plastomes. (**A**) Total number of three-repeat types in all plastomes. (**B**) Number of palindromic repeats by length. (**C**) Number of forward repeats by length. (**D**) Number of tandem repeats by length. (**E**) Pair plot showing the distribution of repeats in *Triticum* species. (**F**) Correlation among different characteristics of *Triticum* plastomes.

In repeat analysis, different forms of repeat sequences in the 21 plastomes, including *T. sphaerococcum* and *T. turgidum* subsp. *durum*, were analyzed. *T. sphaerococcum* had 19 palindromic repeats, 23 forward repeats, and 29 tandem repeats, whereas *T. turgidum* had 21 palindromic repeats, 25 forward repeats, and 27 tandem repeats (Figure 3). Across all species, there was some variation in the number of repeats. The overall number of repeats in these genomes (including palindromic, forward, and tandem repeats) ranged from 59 (*T. monococcum* subsp. *monococcum*) to 75 *T. aestivum* (NC002762), with 71 and 73 repeats found in *T. sphaerococcum* and *T. turgidum* subsp. *durum* respectively. Among these repeats, the highest number of palindromic repeats (21) were detected in four species including *T. turgidum* subsp. *durum* (Figure 3A–E). However, the highest number of forward repeats (26) was detected in the *T. timopheevii* cultivar Tim01 and *T. zhukovskyi* plastomes. Similarly, the highest number of tandem repeats were detected in the *T. aestivum* (NC002762) and *T. turgidum* subsp. *durum* cultivar Langdon plastomes. Overall, there was a positive correlation between plastome size and IR length (Figure 3F). Among these repeats, about 14 and 13 repeats were 15–29 bp in length in *T. sphaerococcum* and *T. turgidum* subsp. *durum*, respectively, while 2 and 4 repeats were 30–44 bp in length, 2 and 2 repeats were 45–59 bp in length, and 1 and 2 repeats were >90 bp in length Similarly, in both plastomes, 18 and 18 forward repeats were 15–29 bp in length, whereas 22 and 20 tandem repeats were also detected 15–29 bp in length (Figure 3A–E).

### 3.4. Simple Sequence Repeat (SSR) Analysis in Triticum Plastomes

**Figure 4:**
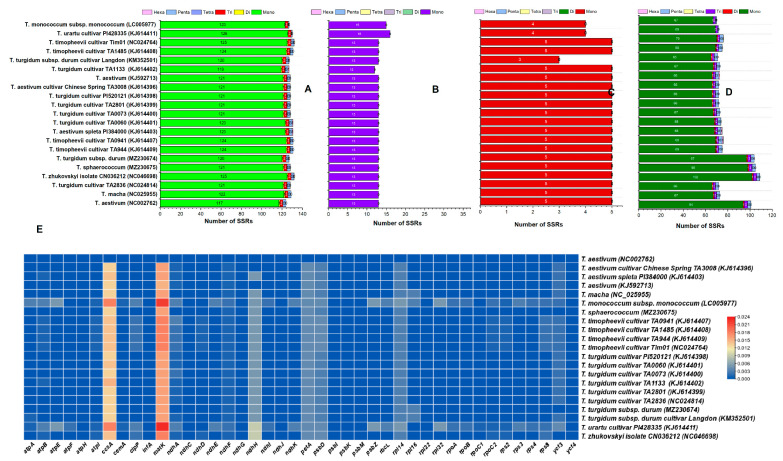
Analysis of simple sequence repeats (SSRs) in the 21 *Triticum* plastomes. (**A**) SSR numbers detected in the 25 species whole plastomes. (**B**) Frequency of identified SSRs in *Triticum* the small single copy (SSC) regions. (**C**) Frequency of identified SSRs in inverted repeat (IR) region. (**D**) Frequency of identified SSRs in the long single copy (LSC) region. (**E**) For *T. turgidum* subsp. *turgidum*, pairwise sequence distance of 42 protein-coding genes shared with the plastomes of related species.

We analyzed perfect SSRs in all studied plastomes (Figure 4). Similar to other plastome characteristics, there were some variations in the number of SSRs in these plastomes: SSR numbers ranged from 124 (*T. aestivum* NC002762) to 132 (*T. timopheevii* cultivar Tim01 and *T. zhukovskyi*). Unexpectedly, SSR numbers also varied even in plastomes of the same species; for example, in *T. aestivum* (NC002762) 124 SSRs were detected whereas 131 were detected in *T. aestivum* (KJ614403). We observed a positive association between SSR numbers and plastome size in *Triticum* (Figure 3F). The *T. sphaerococcum* and *T. turgidum* subsp. *durum* plastomes sequenced in this study contained 128 and 127 SSRs, respectively. In *T. sphaerococcum*, of the 128 SSRs, 121 were mononucleotide repeats (Figure 4A); 1 di, 3 tri, and 3 pentanucleotides were detected; and 82% of SSRs were found in LSC regions, around 10.1% were in SSC regions, and 3.9% were in IR regions. Similarly, *T. turgidum* subsp. *durum* contained 120 mononucleotides, with similar numbers of di, tri, and pentanucleotides to those in the *T. sphaerococcum* plastome, and SSRs present at 81.8% in LSC regions, 10.2% in SSC regions, and 3.9% in IR regions (Figure 4). In all plastomes, the most abundant repeat motifs were mononucleotides, ranging from 117 in *T. aestivum* (NC002762) to 125 in *T. timopheevii* (Tim01), followed by trinucleotides and pentanucleotides, which were next most abundant in most plastomes (Figure 4). Using our search criterion, tetranucleotides and hexanucleotide SSRs were absent in most plastomes except in those of *T. aestivum* spleta PI384000, *T. turgidum* TA0060, and *T. turgidum* cultivar TA1133, in which one hexanucleotide was observed in each plastome. Notably, only one dinucleotide SSR was detected in most plastomes.

### 3.5. Comparative Analysis and Divergence of Triticum Plastomes

Comparison of *T. sphaerococcum* and *T. turgidum* subsp. *durum* with the 19 related plastomes showed that variation existed in whole plastomes. We aligned and compared all plastomes to find the average pairwise distance among the species (Table S2). *T. sphaerococcum* was used a reference in pairwise sequence divergence analysis, in which it showed the highest divergence (0.004) with *T. monococcum* and the lowest divergence with all of *Triticum astivum*, *Triticum trugidum*, and *T. timopheevii* species (0.001) (Table S2). Similarly, the synteny of *T. sphaerococcum* and *T. turgidum* subsp. *durum* plastomes with the 19 plastomes of the other *Triticum* species was analyzed by mVISTA. The results showed high sequence similarities among the plastomes of several species, especially in protein-coding and IR regions (Figure S1). However, divergence was observed in noncoding regions compared with coding regions. The *matK* gene exhibited almost similar divergence in all plastomes. The region between the *rps16* and *psbl* genes showed the highest divergence, except for in *T. sphaerococcum* that had comparatively low divergence. The *atpF* gene exhibited divergence in all cp genomes excluding those of *T. sphaerococcum* and *T. turgidum* subsp. *durum*. Similarly, the noncoding region between the *trnL* and *ndhJ* genes of *T. monococcum* subsp. *monococcum* showed large divergence with the region in *T. turgidum* subsp. *durum* plastomes. Similar results were observed in *psbE* and *petL* regions. Furthermore, a significant divergence was observed in all plastomes between *rpl23-ndhB* regions. Moreover, 42 protein-coding gene sequences were compared to obtain the average pairwise distance among 21 *Triticum* plastomes. Results showed relatively lower levels of average pairwise divergence and various divergent genes, i.e., *matK*, *ccsA*, *ndhH*, *petA*, *psbD*, *rpl14*, and *ycf3*, were detected in these plastomes. The highest pairwise divergence was detected in the *matK* (0.67) and *ccsA* (0.56) genes (Figure 4E).

### 3.6. Evolution and Origin of IRs in Triticum Plastomes

IR regions are considered to be the most conserved regions in a chloroplast genome. Larger plastome sizes correlate with larger IR lengths. Similar to previously described angiosperm plastomes, both the *T. sphaerococcum* and *T. turgidum* subsp. *durum* plastomes also contained IRs with lengths of 20,699 and 20,701 bp, respectively. The gene arrangement in the IR region of these plastomes was more similar to that of *T. aestivum*, in which, seven protein-coding genes (*rpl2*, *rpl23*, *rps12*, *rps7*, *ndhB*, *rps15*, and *ndhH*) are duplicated. Comparative analyses of the plastomes suggested that the smallest IR region was found in *T. turgidum* subsp. *durum* cultivar Langdon (17,066 bp), whereas the largest was detected in all four *T. timopheevii* cultivars (21,553 bp). Furthermore, the IR borders for different genes in the *T. sphaerococcum* and *T. turgidum* subsp. *durum* plastomes were compared with already published plastomes of *Triticum*. Due to the conserved nature of these *Triticum* plastomes no significant changes were observed in four IR junctions (J^LB^, J^SB^, J^SA^, and J^LA^) and genes located across these borders. Although, few variations were detected in some plastomes like location of *ndhH* at J^SA^ is 975 bp in SSC and 207 bp in IRa in all plastomes except in *Triticum turgidum* subsp. *durum* cultivar Langdon which slightly deviating and located 1004 bp in SSC and 208 bp in IRa. (Figure S2). Furthermore, the *rps19* is present in IRa region at J^LA^ of all plastomes but it is located in LSC at J^LA^ in *Triticum turgidum* subsp. *durum* cultivar Langdon and resulted in changing the location of rpl22 gene comparing to all other plastomes. The overall nature of all compared plastomes was found very conserved.

### 3.7. Plastome Phylogenomics and Diversification of Triticum Plastomes

**Figure 5:**
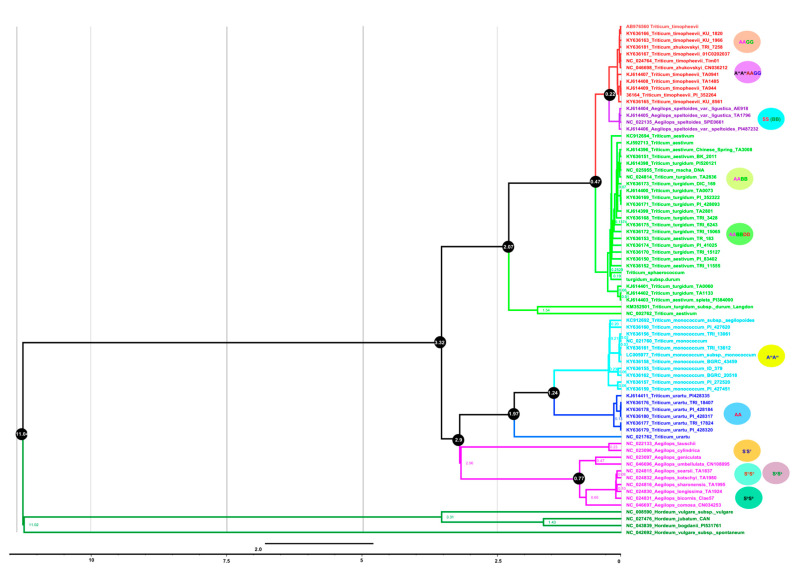
Maximum credible molecular chronogram (time tree) of *Triticum* from BEAST based on whole plastomes, with branch lengths proportional to time and lognormal fossil-based calibrations. The GTR + G substitution model was used with four rate categories and a Yule tree speciation model was applied with a lognormal relaxed clock model in BEAST. Different color branches represent *Triticum* species based on their genomes. The diploid, tetraploid, and hexaploid genomes are shown at the right side of the tree. The 95% highest posterior density credibility intervals are shown for the node ages in black circles (mya). Numbers indicate date estimates for different nodes. A geological time scale is shown at the bottom of the figure.

**Figure 6:**
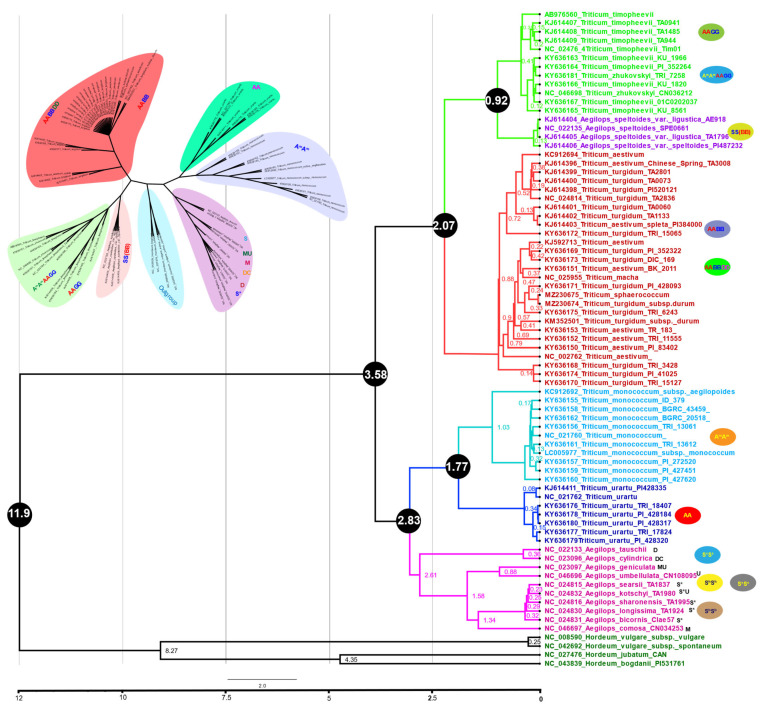
(**A**) Maximum likelihood tree for *Triticum* plastome sequences using 1000 bootstrap repetitions with four outgroup *Hordeum* species: *Hordeum vulgare* subsp. *vulgare*, *Hordeum vulgare* subsp. *spontaneum*, *Hordeum jubatum*, and *Hordeum bogdani*. Different colors represent *Triticum* species based on their genome types. (**B**) Divergence time estimates of Triticeae species based on 42 protein-coding shared genes from Triticeae. The GTR + G substitution model was used with four rate categories and a Yule tree speciation model was applied with a lognormal relaxed clock model in BEAST. Different color branches represent *Triticum* species based on their genomes. The diploid, tetraploid, and hexaploid genomes are shown at the right side of the tree. The 95% highest posterior density credibility intervals are shown for the node ages in black circles (mya). Numbers indicate date estimates for different nodes. A geological time scale is shown at the bottom of the figure.

We used full-length plastome sequences and 42 shared protein-coding concatenated genes among all *Triticum* species to infer the phylogenetic position and divergence time of both *T. sphaerococcum* and *T. turgidum* subsp. *durum* in relation to the other *Triticum* species. Phylogenetic analyses were performed using maximum likelihood (ML) and Bayesian inference methods. Interestingly, the current study provides the first molecular phylogeny of the *Triticum* genus based on the complete plastomes and 42 shared genes from all the *Triticum* species available in the NCBI database. The plastomes of barley (*Hordeum* species) were used as an outgroup for the *Triticum*/*Aegilops* complex. The molecular clock was calibrated using 11.6 million years ago (mya) for the divergence time between barley and wheat (Figure 5 and Figure 6). The topology of these trees is almost identical. According to our phylogenetic analysis, Sitopsis species including S^1^S^1^, S^s^S^s^, S^h^S^h^, and S^b^S^b^ genomes diverged before *T. urartu* (AA) and *T. monococcum* (AA). Similarly, *A. speltoides* (SS) form a monophyletic clade with *T. timopheevii* (AAGG) and *T. zhukovskyi* (A^m^A^m^AAGG), as previously reported [56]. Polyploid *Triticum* species and *A. speltoides* formed a clade indicating that *A. speltoides* is the maternal donor of polypoloid wheat genomes (Figure 5). The precise phylogenetic relationship among the A, B, and D genomes remains a topic for debate. In current results, all hexaploidy *T. aestivum* species form a clade with wild tetraploid *T. turgidum* species based on 60 protein-coding genes. Similar results were observed from whole plastome results except for those from *T. turgidum* subsp. *durum* and *T. aestivum* (NC002762) that both made a separate clade (Figure 6). The divergence time of AB and ABD genomes was estimated at 0.8 to 0.4 mya based on both the complete plastomes and 42 shared genes. The sequenced plastomes of *T. sphaerococcum* and *Triticum T. turgidum* subsp. *durum* shared the same clade and diverged 0.19 to 0.24 mya on both complete plastomes and protein-coding genes (Figure 5 and Figure 6).

## 4. Discussion

In this study, we sequenced two plastomes from the *Triticum* genus, those of *T. sphaerococcum* (ABD) and *T. turgidum* subsp. *durum* (AB), and compared them to 19 complete plastomes that were accessible from GenBank to investigate the relationships within *Triticum*. Both the *T. sphaerococcum* and *T. turgidum* subsp. *durum* plastomes were found to have a quadripartite structure with plastome lengths of 134,531 and 134,015 bp, respectively. Variations were observed in plastome size and GC content compared with those of plastomes from related species, especially with AA genomes. As with previously reported plastomes, variation in plastome length may be due to factors such as loss of IR regions and absence of essential genes [57,58,59]. Diploid (AA), Tetraploid (AB, AG) and hexaploid (ABD, AGA^m^) *Triticum* species plastomes showed conserved gene content and commonly harbored an identical set of annotated unique genes. These plastomes comprised 72–89 protein-coding genes, 4–8 rRNA genes, and 32–42 tRNA genes (Table 1), which are characteristic of plastomes [60,61,62,63]. The number of genes annotated in plastomes ranged from 112 (*T. macha* and *T. monococcum* subs *monococcum*) to 136 (*T. turgidum* subsp. *durum* cultivar Langdon).

Gene transfer and loss commonly occur in plant plastomes [64,65]. For example, *accD*, *ycf1*, *ycf2*, *rpl23*, *rpl22*, *infA*, *rps16*, and *ycf4* were reported partially or completely lost from the legume plastomes [66,67]. Of these, some genes, such as *infA*, have even b plant plastomes een lost multiple times. To the best of our knowledge, this is the first study to use such a large number of representative plastomes to assess gene loss patterns in closely related plants; here, we observed that the *ycf1*, *ycf2*, *ycf15*, and *ycf68* genes were lost from the *Triticum* plastomes that have been sequenced and analyzed (Figure 2). According to our findings the *T. macha* has the highest number of missing protein coding genes comparing to other Triticum plastomes i.e., *ycf1*, *ycf2*, *ycf 15*, *ycf68*, *petB* and *petD* whereas the *T. monococcum* subs *monococcum* and *T. turgidum* subsp. *durum* cultivar Langdon misses one (each) protein coding gene (*ycf1* and *ycf15* respectively) (Figure 2). All these plastomes lacked the *accD* gene, which encodes for a subunit of acetyl-CoA carboxylase. Similar findings have previously been observed in plastomes [59] of Poaceae members. However, this gene has been lost or pseudogenized in several species from Campanulaceae [68,69], Geraniaceae [70], Oleaceae [71], and all Poaceae [59] including the *Triticeae* studied in this work. The deletion of the *accD* gene from chloroplast genomes and its related protein encoded by a nuclear gene in Poaceae perhaps is the first example for a non-ribosomal component [72]. In conclusion, to reduce the size of a chloroplast genome, in addition to gene transfer from the chloroplast to the nucleus, a chloroplast gene could be deleted and a nuclear gene could instead encode the related protein [59].

The remarkable feature of a plastome is the conservation of its most prominent structure and genome size [73]. To determine the contribution of repetitive DNA sequences to genome variation and evolution, we investigated their evolutionary dynamics across these closely related plastomes. The total number of repeats (including palindromic, forward, and tandem repeats) in these genomes ranged from 59 (*T. monococcum*) to 75 (*T. aestivum* (NC002762)) as shown in Figure 3. Overall, there was a positive correlation between the number of repeats and plastome size. In all plastomes, the number of tandem repeats was higher than the number of palindromic and forward repeats. However, the molecular mechanism underlying the de novo generation of novel repeated sequences in a chloroplast genome remains largely unresolved [74]. Numerous repeat numbers have previously been identified in angiosperm plastomes [52,74,75], but the mechanisms underlying the appearance of these tandem repeats remain to be elucidated. Nevertheless, plastome rearrangement, gene expansion, and gene duplication are known to be associated with such repeats [76,77].

Plastome SSR diversity is an attractive research area in plant biology because of SSR’s codominant inheritance, high reproducibility, multiallelic composition, richness, and ease of detection [78,79]. SSRs are normally small tandem mononucleotide repeats, typically found in the chloroplast genome noncoding regions, which usually exhibit intraspecific repeat number differences [80,81]. Genome-wide characterization of SSRs showed that, similar to other plastome characteristics, there was variation in the number of SSRs in these plastomes: SSR numbers ranged from 124 (*T. aestivum* NC002762) to 132 (*T. timopheevii* cultivar Tim01 and *T. zhukovskyi*). The *T. sphaerococcum* and *T. turgidum* subsp. *durum* plastomes sequenced in this study contained 128 and 127 SSRs, respectively (Figure 4). Among these SSR motifs, the most abundant repeat motifs were mononucleotides, ranging from 117 in *T. aestivum* (NC002762) to 126 in *T. urartu* cultivar PI428335, followed by trinucleotides and pentanucleotides. Using our search criterion, tetranucleotide and hexanucleotide SSRs were absent in most plastomes. Similarly, a linear relationship between SSR numbers and plastome size was detected in *Triticum* plastomes. A similar correlation was discovered in previous analysis of various plant whole genomes [79]. As previously reported, genome rearrangement and sequence diversity occur due to an incorrect recombination of these repeat sequences and slipped strand mispairing [82,83]. Furthermore, the occurrence of these repeats indicate that the region is a critical hotspot for the reconfiguration of the plastome genome [83]. Additionally, these repeats represent a useful source for developing genetic markers for *Triticum* species, which could be further applied in phylogenetic and population studies.

Comparative analysis of *Triticum* plastomes showed that analysis of genes with known functions shared 42 protein-coding genes. Furthermore, pairwise sequence divergence analysis shown that *T. sphaerococcum* exhibited the highest divergence with *T. urartu*. Similarly, synteny analysis showed high sequence similarities among the *Triticum* plastomes, especially in the protein-coding and IR regions. The *psbD* and *atpF* genes exhibited divergence in all plastomes excluding *T. sphaerococcum* and *T. turgidum* subsp. *durum*. Similarly, the noncoding region between the *trnL-ndhJ* gene of *T. monococcum* subsp. *monococcum* was significantly divergent from that of the *T. turgidum* subsp. *durum* plastomes. Similar results were observed for *psbE* and *petL* regions. Furthermore, a significant divergence was observed in all plastomes between the *rpl23-ndhB* regions. Moreover, the average pairwise distances among 42 shared protein-coding genes revealed that the most divergent genes were *matK*, *ccsA*, *ndhH*, *petA*, *psbD*, *rpl14*, and *ycf3* in almost all species. This is in agreement with previously reported findings for the plastomes of angiosperms [33,52]. Menezes, et al. [84] concluded that divergent plastome genes are predominantly detected in the LSC regions, suggesting a more rapid evolution trend [85].

In addition to the overall conservation of gene content, the analyzed *Triticum* plastomes were structurally conserved and they displayed an almost uniform order of the same set of genes with slight differences at boundary junctions. The plastomes of *Triticum* in general are smaller (like other Poaceae members) [59] than most angiosperms due to the pseudogenization of the *ycf1* and *ycf2* genes, i.e., two of the longest open reading frames in angiosperms. Due to the conserved nature of these Triticum plastomes, no substantial changes in four IR junctions and gene positions across IR boundaries were identified, with only minor variations between plastomes. This is not exceptional, as a slight IR expansion/contraction has also been reported in other angiosperm plastomes [71,86,87].

Despite a plethora of molecular phylogenies, it remains a lack of understanding of the relationships among the *Triticum* genus [20,22,23,88,89,90,91,92]. The acceptance levels of taxa vary greatly among studies at the genus level and below [20,93,94]. One important reason for this is the complex mode of evolution within Triticeae. The majority of species are allopolyploids and many of them likely originated repeatedly with the process involving genetically different parent species [95,96,97]. Bread wheat is the most prominent polyploid and evolved via consecutive hybridizations of three diploids; thus, it combines three related genomes (A, B, and D) [11,92]. It is assumed that diploid species and monogenomic taxa are the basic units within Triticeae and that the heterogenomic polyploids form a second level of taxonomic entities [98,99]. The paternal ancestors of polyploid wheat, i.e., *T. urartu*, *T. monococcum*, and *A. tauschii*, have been well established based on the sequence analysis of many nuclear genes. The history of the maternal ancestors of the G genome of *Timopheevi* and the B genome of Emmer wheat has been more difficult to decipher based on nuclear genome sequences.

We presented phylogenetic analyses of *Triticum* based on complete plastomes and 42 concatenated protein-coding genes shared among these *Triticum* species. Our results are similar to those of previous studies and in particular the divergence of *H. vulgare* from wheat, which was estimated previously to have occurred approximately 8–12 mya [53,56,100] is 11.04 and 11.9 mya using complete plastomes and protein-coding genes, respectively. These values are in the range of estimates reported by [53] (approx. 7–16 mya) and [100] (8.3–11.3 mya), but more similar to the 11.4 ± 0.6 mya reported by [56]. Similarly, of particular interest to us was the precise phylogeny and dating of the age of the wheat genome donors. Our analysis indicates that the donors (or their close relatives) of the wheat genomes diverged within the past 3 million years as previously reported [101]. According to our phylogenetic analysis, Sitopsis species, including S^1^S^1^, S^s^S^s^, S^h^S^h^, and S^b^S^b^ genomes, diverged before *T. urartu* (AA) and *T. monococcum* (AA) around 2.8–2.9 mya. Our estimates are in the range of previous reports [53,56], which ranged rather widely from 2 to 6 mya. Similarly, *T. monococcum* diverged from *T. urartu* about 1.7–1.9 mya. These results are more recent than the previous estimates [53,56]. Furthermore, our results showed that polyploid *Triticum* species and *A. speltoides* formed a clade indicating that *A. speltoides* is the maternal donor of polyploid wheat genomes and diverged ~0.2–0.9 mya based on complete plastomes and protein-coding gene trees, respectively, from *T. timopheevii* (AABB) and *T. zhukovskyi* (GG) genomes. Similar results were reported previously by various researchers [22,53,101]. The relationships within this clade support the hypothesis that two different *A. speltoides* lineages were involved in their formation [5,9,22,102]. As in previous studies, the direct maternal donor for *T. timopheevii* and *T. zhukovskyi* (G) were identifiable because they share the chloroplast haplotype of *A. speltoides* genomes. According to our results, the donor remains uncertain for *T. turgidum* and *T. aestivum* (B), indicating that either our sampling of *A. speltoides* was insufficient to cover the species diversity or that a now extinct donor lineage previously existed. *Aegilops speltoides* and the polyploid wheat species form three groups: (1) most *A. speltoides* accessions form a clade of their own (S), (2) they share a clade with *T. timopheevii* and *T. zhukovskyi* wheat, and (3) all accessions of *T. turgidum* and *T. aestivum* share the same haplotype (B). Additionally, the usage of entire plastomes and shared protein-coding genes also suggests that diploid *Triticum* species (A) diverged from D-genome taxa and the remaining *Aegilops* species 2.83 mya.

## 5. Conclusions

Decoding *Triticum* plastomes during the last three decades has greatly increased levels of available plastomic data and provided an improved picture of *Triticum* plastome evolution. The elucidated plastomes of diploid (AA), tetraploids (AB, AG) and hexaploids (ABD, AGAm) show conserved gene content. Despite significant effort to determine *Triticum* plastomes at the genus/species level, certain species remain poorly studied; thus, full systematic phylogenetic studies are lacking. Based on 42 shared genes and full plastomes, the tree height between *H. vulgare* and *Triticum* was found to be 11.04 and 11.9 mya, respectively. In the future, additional plastomes will be sequenced and comparatively analyzed to provide a more complete picture of *Triticum* plastome evolution.

## Figures and Tables

**Table 1:** Summary of the genome features of complete *Triticum* plastomes.

	Size (bp)	GC (%)	LSC (bp)	SSC (bp)	IR (bp)	PCD (bp)	tRNA (bp)	rRNA (bp)	Gene (n)	PCD (n)	tRNA (n)	Accesion No.
*T. sphaerococcum*	134,531	38.3	80,342	12,791	20,699	59,538	3004	9192	131	84	39	MZ230675
*T. turgidum* subsp. *durum*	134,015	38.3	79,817	12,788	20,701	59,538	3004	9192	131	84	39	MZ230674
*T*. aestivum (NC_002762)	134,545	38.3	80,348	12,790	20,703	59,946	3264	9191	133	83	42	NC002762
*T. aestivum spleta* PI384000	135,919	38.3	56,296	13,144	21,541	58,701	2469	9192	123	82	33	KJ614403
*T. aestivum* (KJ592713)	133,873	38.3	56,228	12,791	20,573	59,664	2943	9192	131	84	39	KJ592713
*T. aestivum* cultivar Chinese Spring TA3008	135,835	38.3	56,254	12,792	21,541	58,683	2469	9192	123	82	33	KJ614396
*T. macha*	135,899	38.3	56,298	12,791	21,522	54,723	2480	9190	112	72	32	NC_025955
*T. monococcum* subsp. *monococcum*	136,886	38.3	56,492	12,806	21,547	54,723	2480	9192	112	72	32	LC005977
*T. timopheevii* cultivar Tim01	136,157	38.3	56,550	12,795	21,553	58,686	2469	9192	123	82	33	NC024764
*T. timopheevii* cultivar TA0941	136,074	38.3	56,474	12,789	21,553	58,686	2469	9192	123	82	33	KJ614407
*T. timopheevii* cultivar TA944	136,124	38.3	56,523	12,789	21,553	58,686	2469	9192	123	82	33	KJ614409
*T. timopheevii* cultivar TA1485	136,119	38.3	56,518	12,789	21,553	58,686	2469	9192	123	82	33	KJ614408
*T. turgidum* cultivar TA0060	135,926	38.3	56,284	12,792	21,541	58,701	2469	9192	123	82	33	KJ614401
*T. turgidum* cultivar TA0073	135,865	38.3	56,266	12,792	21,541	58,683	2469	9192	123	82	33	KJ614400
*T. turgidum* cultivar TA1133	135,889	38.3	56,287	12,793	21,541	58,701	2469	9192	123	82	33	KJ614402
*T. turgidum* cultivar TA2801	135,835	38.3	56,254	12,792	21,541	85,683	2469	9192	123	82	33	KJ614399
*T. turgidum* cultivar TA2836	135,835	38.3	56,254	12,792	21,541	58,683	2469	9192	123	82	33	NC_024814
*T. turgidum* cultivar PI520121	135,836	38.3	56,255	12,792	21,541	58,683	2469	9192	123	82	33	KJ614398
*T. turgidum* subsp. *durum* cultivar Langdon	135,948	38.3	56,293	15,992	17,066	61,746	2913	9192	136	89	39	KM352501
*T. urartu* cultivar PI428335	136,865	38.3	56,454	12,824	21,547	58,683	2469	9192	123	82	33	KJ614411
*T. zhukovskyi* isolate CN036212	136,028	38.3	80,257	12,790	21,495	59,208	2871	9061	128	82	38	NC_046698

**Table 2:** Gene composition in *T. sphaerococcum* and *T. turgidum* subsp. *durum* plastomes.

Category of Genes	Group of Genes	Name of Genes
Genes for photosynthesis	Subunits of ATP synthase	*atpA*, *atpB*, *atpE*, *atpF*, *atpH*, *atpI*
	Subunits of photosystem II	*psbA*, *psbB*, *psbC*, *psbD*, *psbE*, *psbF*, *psbI*, *psbJ*, *psbK*, *psbL*, *psbM*, *psbN*, *psbT*, *psbZ*, *ycf3*
	Subunits of NADH-dehydrogenase	*ndhA*, ndhB*, *ndhC*, *ndhD*, *ndhE*, *ndhF*, *ndhG*, *ndhH*, *ndhI*, *ndhJ*, *ndhK*
	Subunits of cytochrome b/f complex	*petA*, *petB*, *petD*, *petG*, *petL*, *petN*
	Subunits of photosystem I	*psaA*, *psaB*, *psaC*, *psaI*, *psaJ*
	Subunit of rubisco	*rbcL*
Self-replication	Large subunit of ribosome	*rpl14*, *rpl16*, rpl2*, *rpl20*, *rpl22*, rpl23*, *rpl32*, *rpl33*, *rpl36*
	DNA dependent RNA polymerase	*rpoA*, *rpoB*, *rpoC1*, *rpoC2*
	Small subunit of ribosome	*rps11*, rps12*, *rps14*, rps15*, *rps16*, *rps18*, rps19*, *rps2*, *rps3*, *rps3*, *rps4*, rps7*, *rps8*
Other genes	c-type cytochrom synthesis gene	ccsA
	Envelop membrane protein	*cemA*
	Protease	*clpP*
	Translational initiation factor	*infA*
	Maturase	*matK*
Unknown	Conserved open reading frames	*ycf4*

* represent duplicated genes.

**Table 3:** The genes with introns in the *T. sphaerococcum* and *T. turgidum* subsp. *durum* plastomes and the length of exons and introns.

Gene	Strand	ExonI		IntronI		ExonII		IntronII		ExonIII	
		T.S	T.T	T.S	T.T	T.S	T.T	T.S	T.T	T.S	T.T
*trnK*-UUU	−	38	38	2486	2490	36	36				
*rps16*	−	40	40	843	845	218	218				
*trnS*-CGA	−	31	31	658	658	62	62				
*atpF*	+	145	145	825	825	407	407				
*ycf3*	−	124	124	756	756	230	230	731	731	159	159
*rps12*	+	114	114			223	223	540	540	32	32
*trnL*-UAA	+	36	36	588	588	51	51				
*trnV*-UAC	−	38	38	581	581	53	53				
*petB*	+	6	6	748	748	642	642				
*petD*	+	8	8	749	749	475	475				
*rpl16*	−	9	9	1043	1043	402	402				
rpl2 *	−	385	388	663	663	431	431				
ndhB *	−	775	775	712	712	758	758				
*trnT*-CGU	+	32	32	787	787	59	59				
*trnA*-UGC	+	37	37	805	805	36	36				
*ndhA*	−	550	550	1036	1036	539	539				
*trnA*-UGC	−	37	37	805	805	36	36				
*trnT*-CGU	−	32	32	787	787	59	59				

* Represent duplicated genes. *T. sphaerococcum* = (T.S), *T. turgidum* subsp. *durum* = (T.T).

## Data Availability

All the 75 plastomes are available at the NCBI database.

## Supplementary Materials

The following are available online at https://www.mdpi.com/article/10.3390/ijms23052783/s1.
