# Supplementary figures and images for: The Plastome Sequences of Triticum sphaerococcum (ABD) and Triticum turgidum subsp. durum (AB) Exhibit Evolutionary Changes, Structural Characterization, Comparative Analysis, Phylogenomics and Time Divergence
Source: Int J Mol Sci. 2022 Mar 3;23(5):2783. doi: 10.3390/ijms23052783 (PMC8911259; doi:10.3390/ijms23052783)

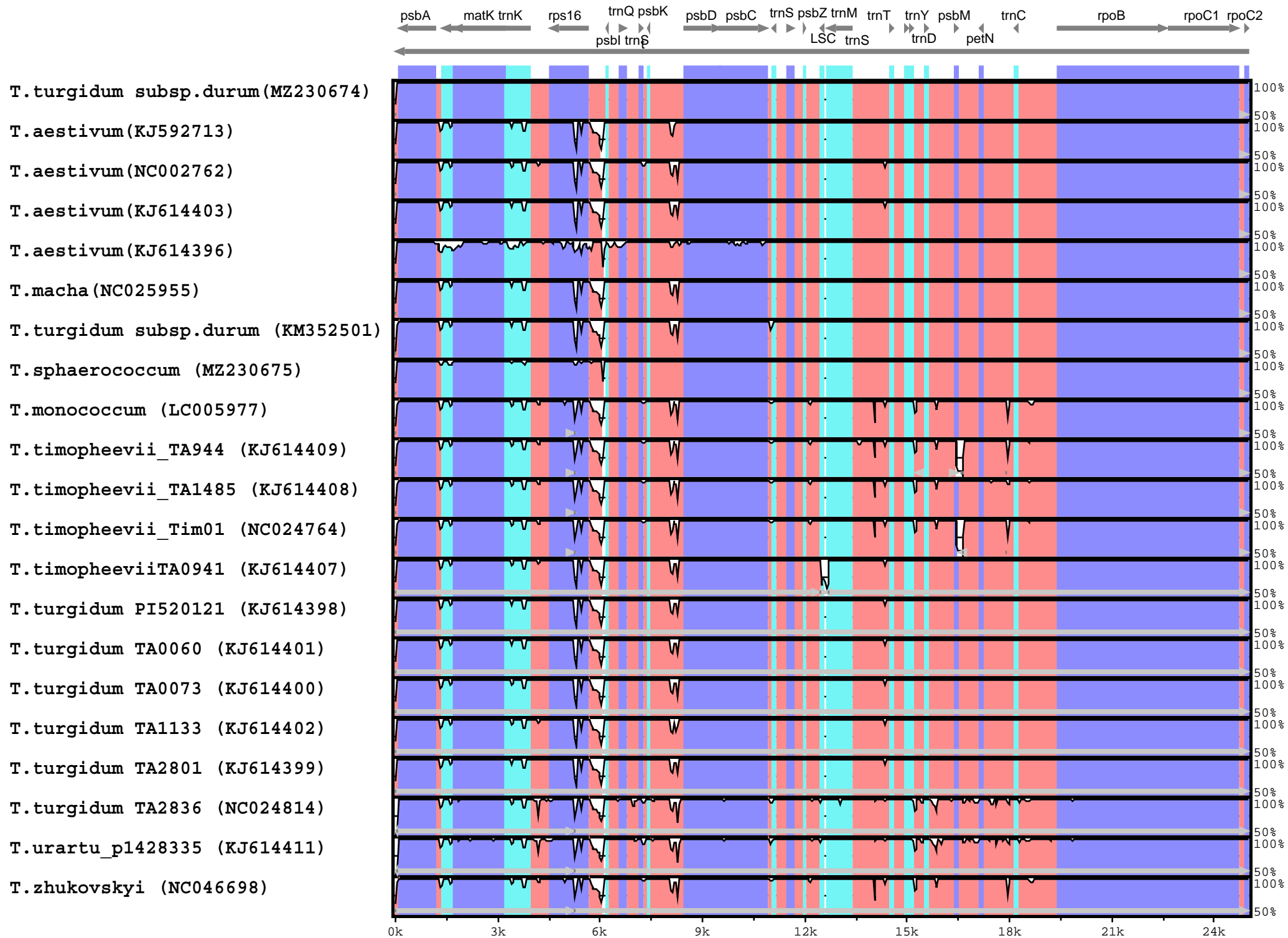

→ contig  
→ gene  
■ exon  
■ UTR  
■ CNS  
■ mRNA

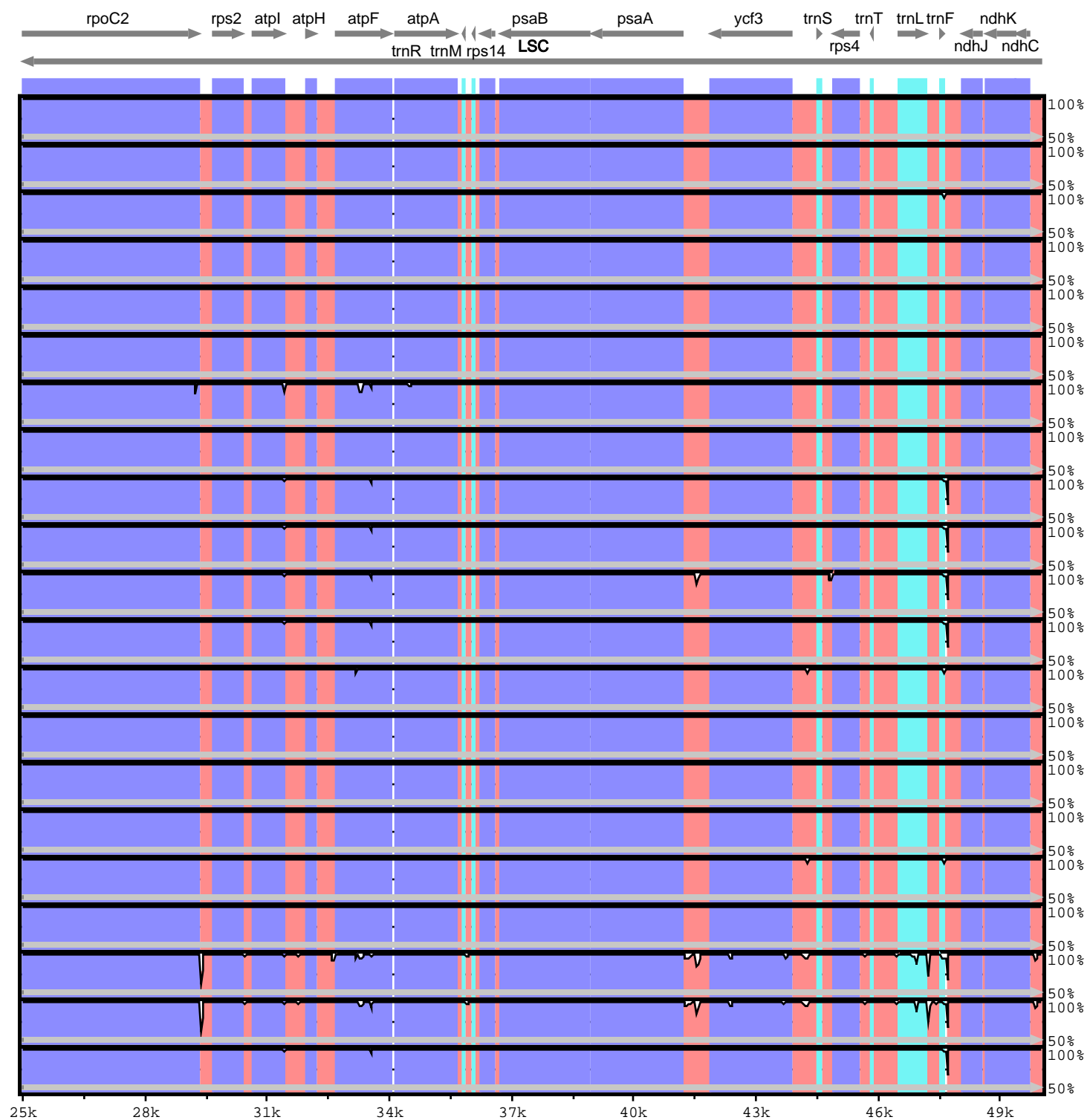

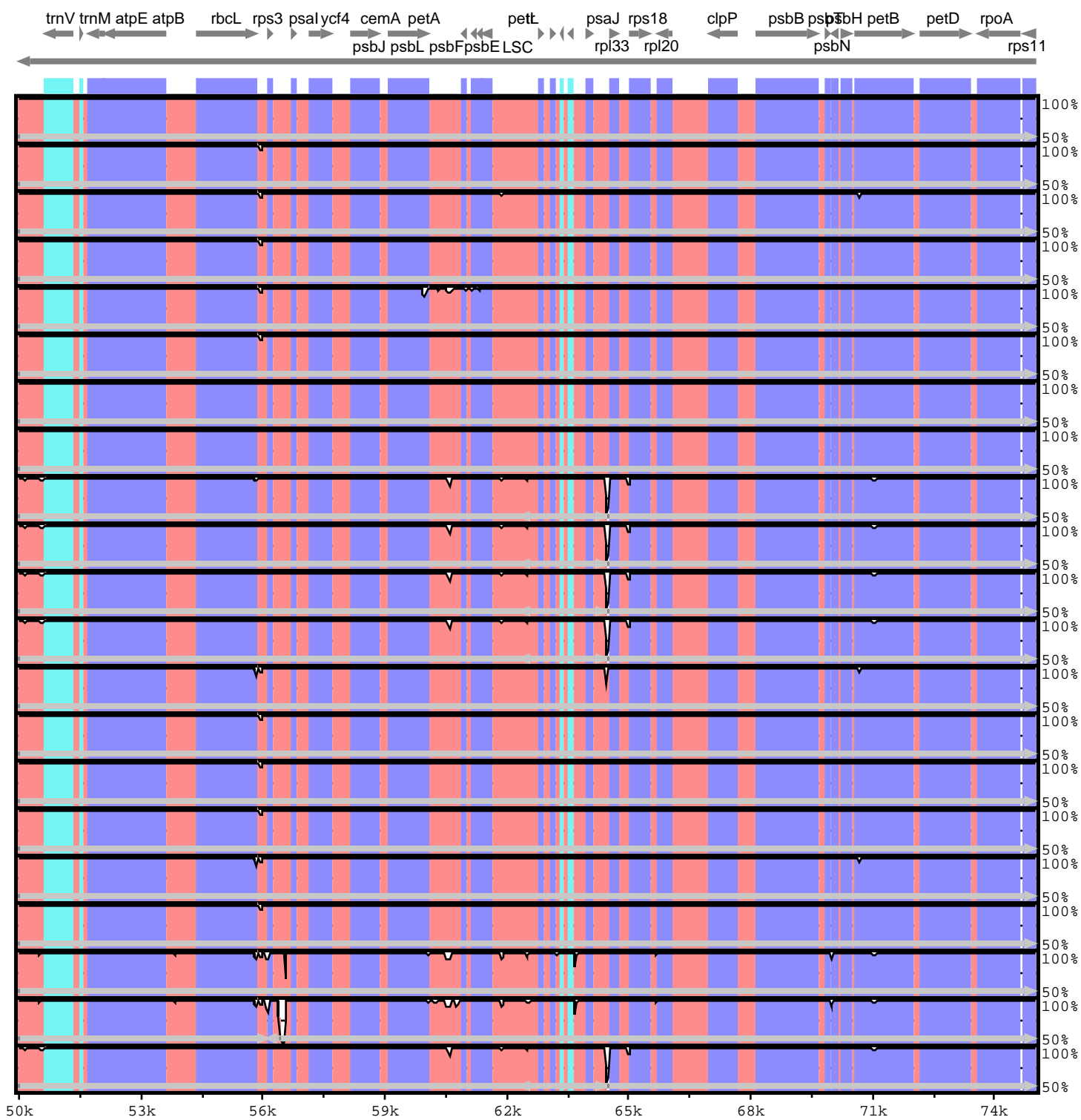

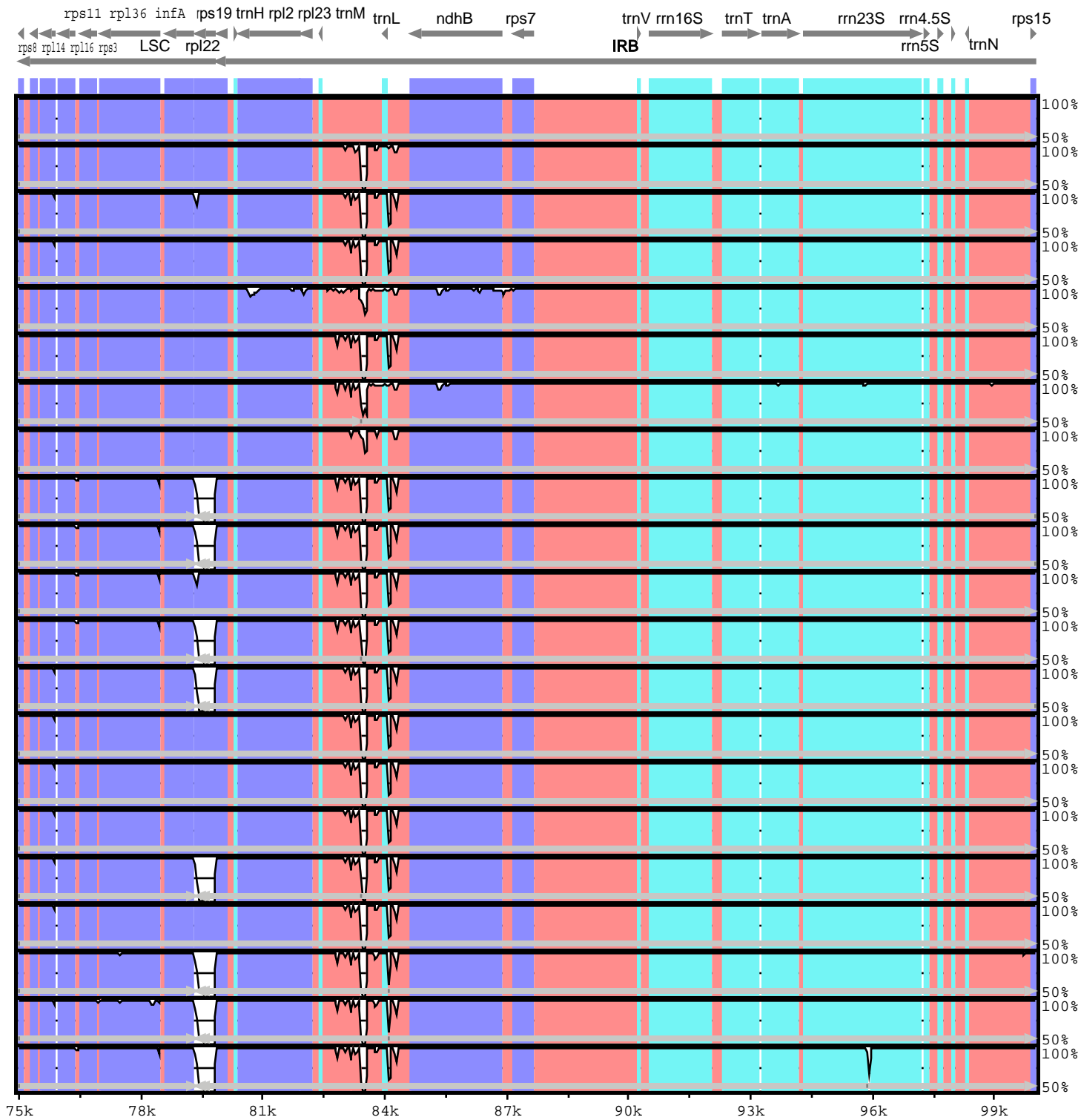

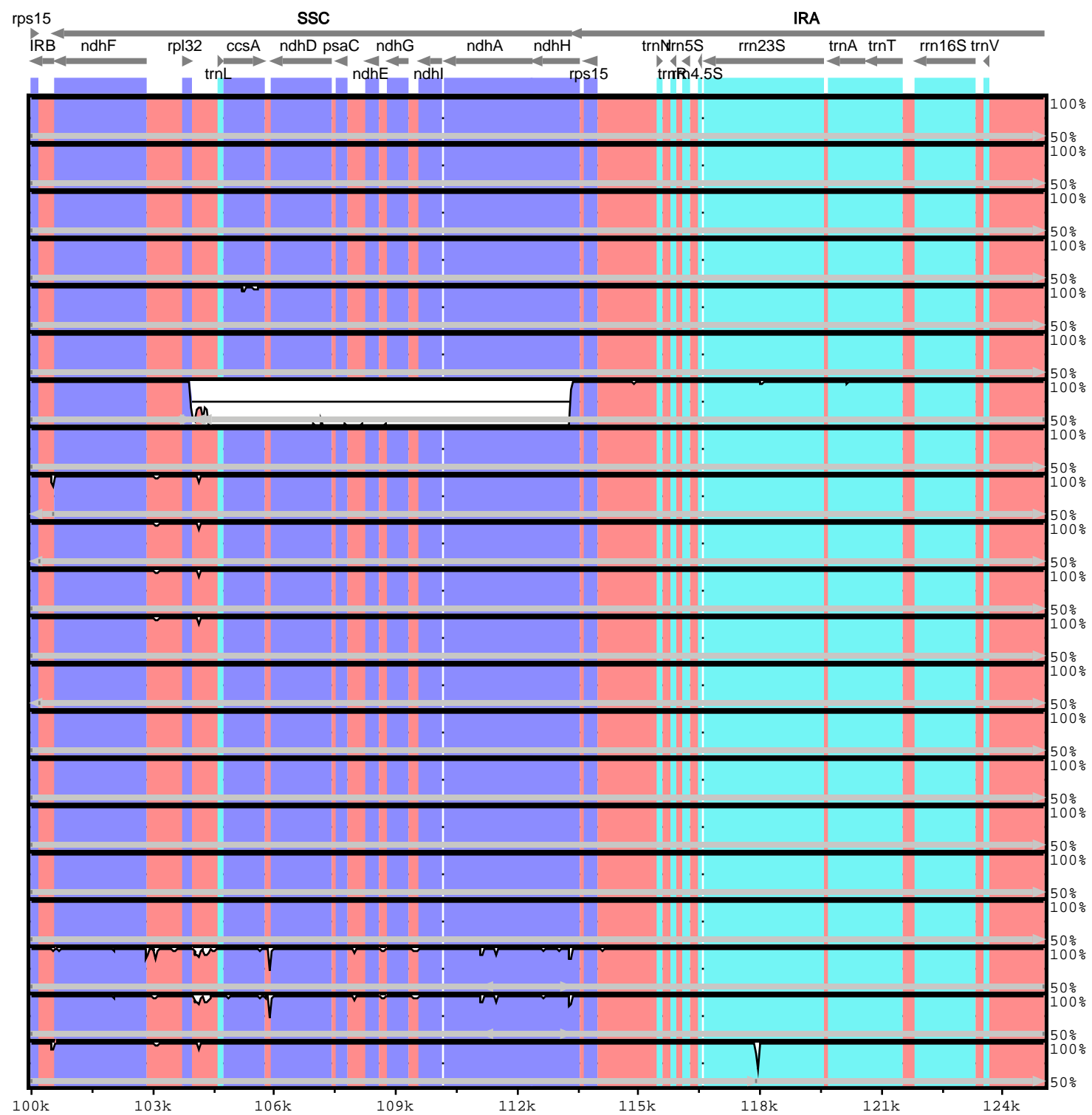

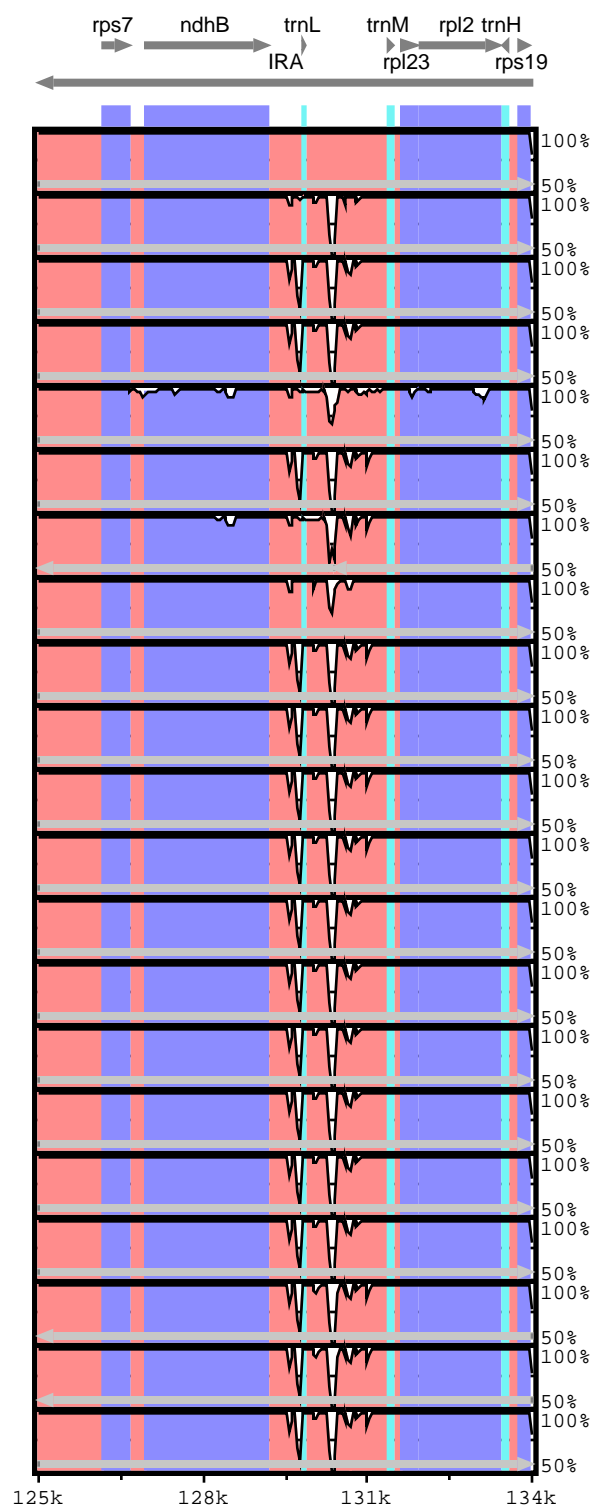

Supplement: Supplementary file 1 [file ijms-23-02783-s001.zip › Figure S1.pdf]

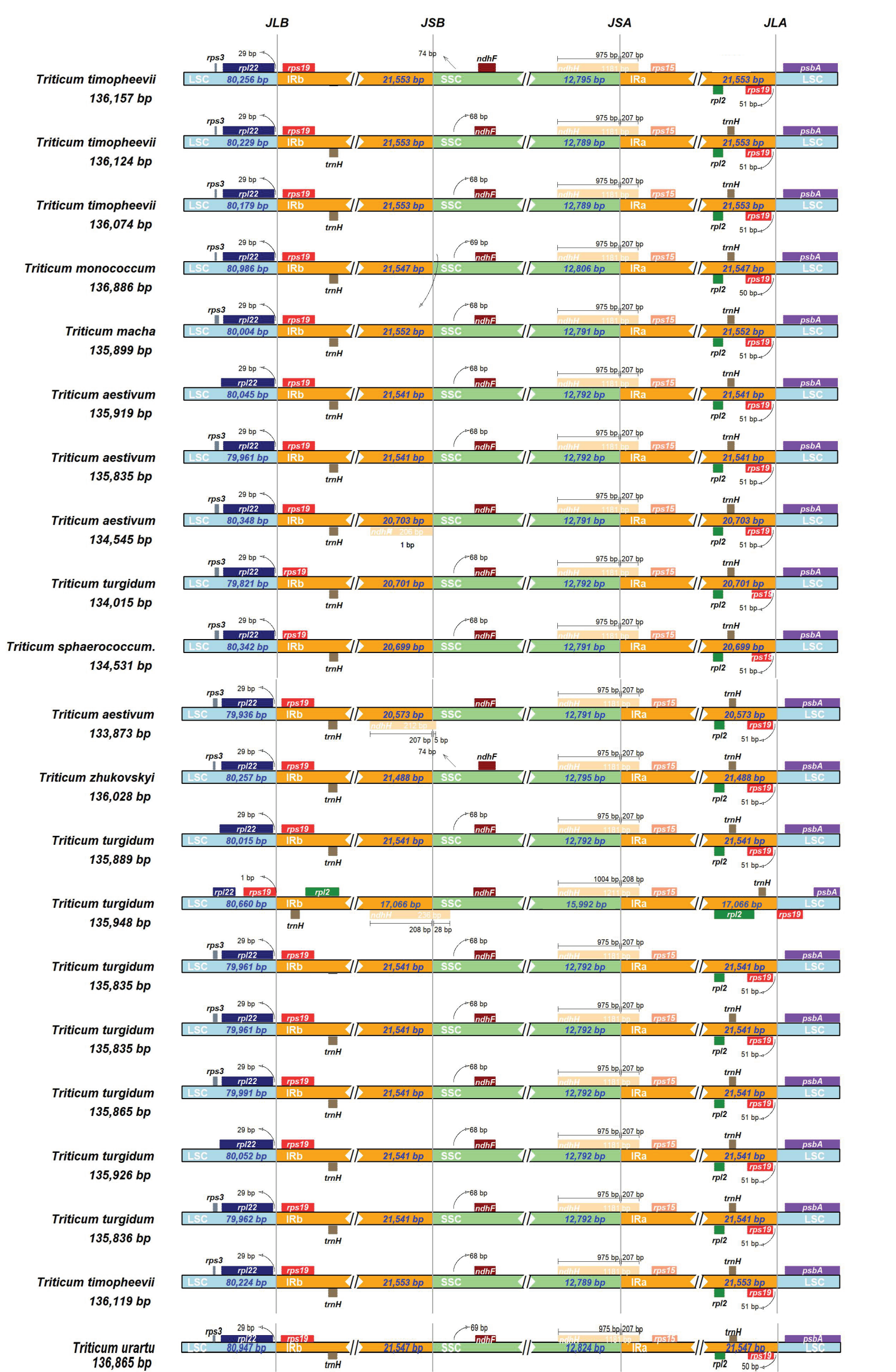

Supplement: Supplementary file 1 [file ijms-23-02783-s001.zip › Figure S2.pdf]
